# Integrated System for Monitoring Muscular States during Elbow Flexor Resistance Training in Bedridden Patients

**DOI:** 10.1155/2019/4290957

**Published:** 2019-01-17

**Authors:** Taojin Xu, Zhongwei Jiang, Jongyeob Jeong, Minoru Morita, Hongbin Xu

**Affiliations:** ^1^Micro Mechatronics Laboratory, Graduate School of Science and Engineering, Yamaguchi University, 2-16-1 Tokiwadai, Ube 755-8611, Japan; ^2^School of Mechanical Engineering, Chongqing University of Technology, No. 69 Hongguang Avenue, Banan District, Chongqing 400054, China

## Abstract

To improve or maintain the physical function of bedridden patients, appropriate and effective exercises are required during the patient's bed rest. Resistance training (RT) is an effective exercise for improving the physical function of bedridden patients, and the improvement of the physical function is caused by mechanical stimuli associated with RT. Currently, the measured mechanical stimuli are external variables which represent the synthetic effect of multiple muscles and body movements. Important features of stimuli experienced by muscles are of crucial importance in explaining muscular strength and power adaptation. This study describes an integrated system for assessing muscular states during elbow flexor resistance training in bedridden patients, and some experiments were carried out to test and evaluate this system. The integrated system incorporates an elbow joint angle estimation model (EJAEM), a musculoskeletal model (MSM), and a muscle-tendon model. The EJAEM enables real-time interaction between patient and MSM. The MSM is a three-dimensional model of the upper extremity, including major muscles that make up the elbow flexor and extensor, and was built based on public data. One set of concentric and eccentric contraction was performed by a healthy subject, and the results of the calculations were analyzed to show important features of mechanical stimuli experienced by muscles during the training. The integrated system provides a considerable method to monitor the body-level and muscle-level mechanical stimuli during elbow flexor resistance training in bedridden patients.

## 1. Introduction

Patients are confined to bed as a consequence of illness, aging, and major surgery. Data from the Ministry of Health, Labour and Welfare show that there are approximately 1.7 million bedridden older adults in 2010 in Japan and this number will increases to 2.3 million in 2025 [1]. The prolonged bedridden behavior of patients will exacerbate skeletal muscle wasting and consequently results in the decline of physical function [2, 3]. The decreased physical function increases the patient's dependence on bed rest, which in turn exacerbates the patient's condition. In order to reverse this exacerbation, appropriate and effective exercises are required during the patient's bed rest [4].

Many studies provided evidence that the resistance training (RT) is an effective method for improving physical function of the bedridden patient [5–7]. The RT refers to the exercise that causes the muscles to contract against an external resistance for the purpose to increase muscular strength and power. During RT, it is thought that the improvement of muscular strength and power is caused by mechanical stimuli, which are related to the kinematic and kinetic variables associated with RT (e.g., force, velocity, power, and work) [8].

Currently, these kinematic and kinetic variables are usually measured by using equipment such as dynamometers, linear position transducers, and force plates [9–11]. Force, displacement, and velocity are measured by using a force plate and linear position sensors. Power is calculated by sampling the system pressure or mechanically defined as the product of force and velocity. Work is calculated as the product of force and displacement. The measured force, velocity, and power are external variables which represent the synthetic effect of multiple muscles and body movements. To obtain a better appreciation of how mechanical stimuli affect strength and power adaptation, details of the training (such as details of the movement and the way how the external resistance load is applied to the body), and important characteristics of mechanical stimuli associated with the training are of crucial importance. The different ways of moving or lifting the load will have varied effects on the strength and power adaptations [8]. And only the maximum or average value of the load is not sufficient to evaluate the training effect. Moreover, aging or illness is usually accompanied by changes in the muscle's morphology and architecture (e.g., sarcopenia usually occurs with decrease in muscle mass) [12]. The appropriate volume of training for these patients is different from that of healthy people. Therefore, a scientific method is needed to quantify these differences and to see important features of mechanical stimuli during the training.

Musculoskeletal modeling is a powerful tool to research the mechanical behavior of human muscles by using the methods of mechanics [13]. This method quantifies the mechanical and physiological properties of each muscle through parametric modeling, enabling researchers to quantify muscle differences and gain insight into the states (usually refer to mechanical states such as muscle-tendon length changes, muscle fiber force, and velocity) of each muscle during the movement. In this paper, we present the concept of using musculoskeletal modeling as a methodology to estimate muscular states during RT for bedridden patients. Currently, a number of researchers use musculoskeletal modeling to study the influence of muscle intrinsic properties on sports performance of athletes such as running [14, 15] and jumping [16, 17], but few have discussed its application in RT for bedridden patients.

We searched many physical therapies recommended to people with injuries and disabilities [18] and chose the elbow flexor RT for bedridden patient. This exercise focuses on the development or maintenance of flexor strength of the upper extremity, and it is simple and applicable for many circumstances such as hospitals, rehabilitation centers, and homes. In hemodialysis patients, doctors often use it to avoid the sedentary lifestyle of patients and improve their fitness status before and after kidney transplantation [19]. Moreover, the elbow flexor RT is an important exercise in pulmonary rehabilitation [20]. Arm training will result in a significant increase in oxygen intake and exercise dyspnea, ultimately increasing arm endurance, regulating dynamic over-inflation, and reducing symptoms in patients with chronic obstructive pulmonary disease [21]. More importantly, an improvement in muscle strength will lead to an improvement in physical function of the upper limbs, such as reaching or lifting an object, and ultimately reversing the patient's dependence on bed rest [7]. For this kind of RT, this study established an integrated system to estimate the muscular states during the training. The remainder of the paper is structured as follows: Section 2 describes the design concepts of the system, details of the measurement, and analysis methods; Section 3 presents some experimental results. A brief discussion was made in Section 4.

## 2. Measurements and Analysis Methods

### 2.1. Design Concepts of the System

**Figure 1:**
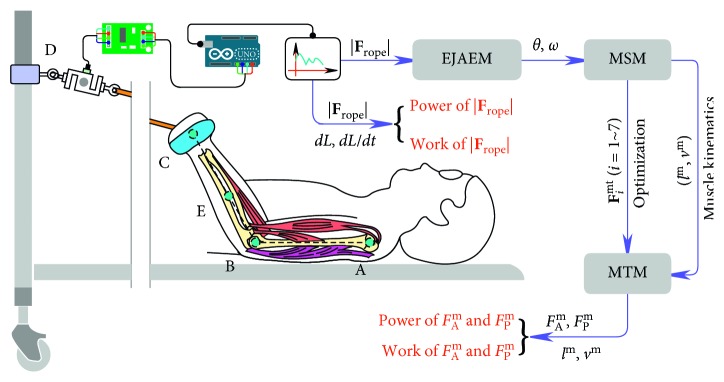
Design concepts of the system and data flow in estimation. *θ* is the elbow joint angle, and *ω* is the angle velocity. *|* **F**_rope_ *|* is the resistance force. *dL* and *dL*/*dt* are the length change and change rate of the TheraBand. *F*_A_^m^ and *F*_P_^m^ are the active muscle fiber force and passive muscle fiber force. *l* ^m^ and *v* ^m^ are the muscle fiber length and velocity. **F**_*i*_^mt^ is the optimized MTF. *|* **F**_rope_ *|* represents mechanical stimuli measured at the body level, and the *F*_A_^m^, *F*_P_^m^, *l* ^m^, and *v* ^m^ represent mechanical stimuli estimated at the muscle level.

Figure 1 illustrates the design concepts of the system. For simplicity of the system, we only use one load cell to measure the time-varying resistance force during the training. The measured resistance force is an external variable, and its resulting power and work do not contain details of stimuli experienced by muscles. Therefore, we established an elbow joint angle estimation model (EJAEM), a musculoskeletal model (MSM), and a muscle-tendon model (MTM) to estimate muscular states during the training. The EJAEM serves as an analytical description of the experimental setup, and it enables real-time interaction between patient and MSM. The MSM is a three-dimensional model of the upper extremity, including major muscles that make up the elbow flexor and extensor and was built based on public data [22, 23]. The MSM provides the kinematics and kinetics required in optimization of muscle-tendon force (MTF) and estimation of muscular states. The MTM was established to estimate the active and passive muscle fiber force for the reason that the optimized MTF is a resultant force of active and passive muscle fiber force, and the power of active muscle fiber force is meaningful for evaluation of muscles.

**Figure 2:**
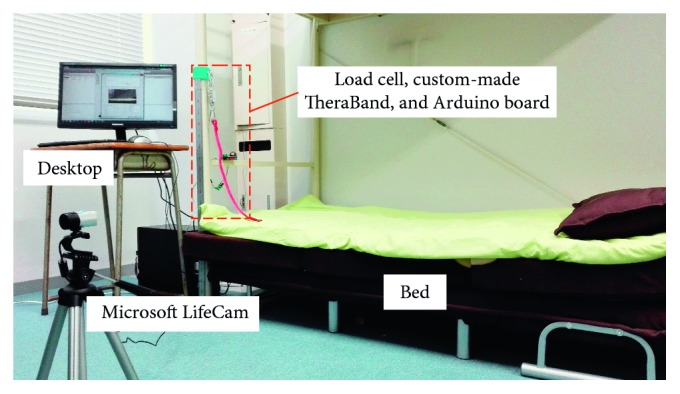
Layout of the experimental setup.

Figure 2 shows the layout of the experimental setup. In the training, the patient is positioned in a bed in supine with his forearm flexing or extending to oppose resistance force produced by a custom-made TheraBand. TheraBand is connected to a load cell which is anchored to the bed by a lifting hook and is utilized to record the resistance force posed by the TheraBand. The force data are converted into a digital signal by an A/D converter and sent to a desktop using an Arduino board. A web camera is utilized to record video of the forearm movement, and the recorded video was used to calculate real elbow joint angle for the testing of the measuring system.

### 2.2. The Elbow Joint Angle Estimation Model

**Figure 3:**
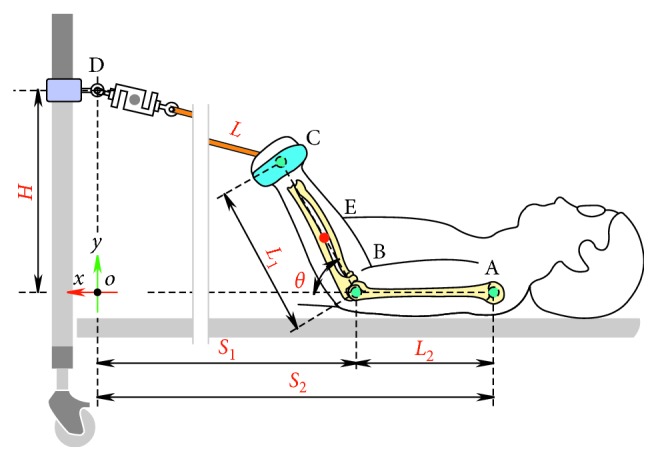
Physical model used to estimate the elbow joint angle and the geometric parameters and coordinates of the experimental setup. The model is a simplified two-dimensional model in the sagittal plane.

The EJAEM plays an essential role in the kinematic and kinetic analysis of forearm movement.  Figure 3 illustrates the physical model used to estimate elbow joint angle, and it includes the coordinates and geometrical parameters about the training setup. In the model, *L*, *L*_1_, and *L*_2_ denote the length of TheraBand, forearm, and upper arm. *S*_1_ and *S*_2_ represent the *x* coordinate of the elbow and shoulder joint. *A*, *B*, *C*, and *E* denote the position of the shoulder, elbow, hand, and center of gravity. One end of the load cell is connected to the bed at point *D* by using a lifting hook, and the other end is connected to the TheraBand. *θ* denotes the elbow joint angle.

**Figure 4:**
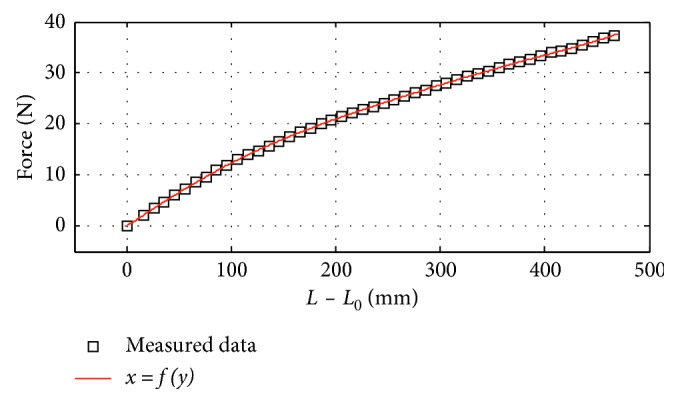
Load versus length-change curve of the custom-made TheraBand. *L*_0_ is the initial length of the TheraBand.

As we can see from the physical model, with specific *H*, *S*_1_, *S*_2_, *L*_1_, and *L*_2_, *θ* is closely related to the current length of the TheraBand *L*. This implies that if we know *L*, we can predict the elbow joint angle through some simple geometric calculation. As shown in Figure 4, we stretched the TheraBand to a series of lengths and recorded the force data to obtain its load versus length-change curve.

Figure 4 shows a strong one-to-one relationship between force and length. We use a polynomial equation to approximate the nonlinear relationship between load and length as follows:

(1) $$\begin{array}{c} L=\Psi_{1}\left(\left|F_{\text{rope}}\right|\right), \end{array}$$

where *ψ*_1_(*x*) is the polynomial equation of *x* and its expression is different for different custom-made TheraBands. |**F**_rope_| is the force data recorded from the load cell and is the norm of **F**_rope_.

According to the physical model, *L* is a trigonometric function of *θ*, and its mathematical expression can be expressed as follows:

(2) $$\begin{array}{c} {\left(H-L_{1}\sin\theta \right)}^{2}+{\left(S_{1}-L_{1}\cos\theta \right)}^{2}=L^{2}. \end{array}$$

Because the TheraBand has an initial length *L*_0_ and resistance force is 0 when the TheraBand length is less than *L*_0_, the elbow joint angle estimated by EJAEM is never less than the initial angle *θ*_0_. According to (1) and (2) and the recorded |**F**_rope_|, we eventually get *θ* as a function of time as follows:

(3) $$\begin{array}{c} \theta \left(t\right)=\Psi_{2}\left(\left|F_{\text{rope}}\right|\right)=\Psi_{3}\left(t\right). \end{array}$$

Furthermore, we can get the angular velocity *ω* as follows:

(4) $$\begin{array}{c} \omega \left(t\right)=\frac{d\theta \left(t\right)}{dt}=\frac{\Psi_{3}\left(t+dt\right)-\Psi_{3}\left(t\right)}{dt}. \end{array}$$

### 2.3. Musculoskeletal Model

**Figure 5:**
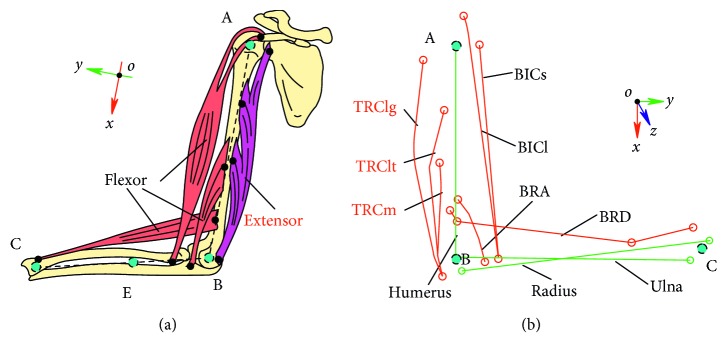
(a) Extensor and flexor of the human upper limb in the sagittal plane. (b) Three-dimensional MSM illustrates bones (green lines) and muscles (red lines) across elbow joint.

The elbow flexor RT incorporates concentric and eccentric movements in which flexor and extensor are dominant. According to the anatomical descriptions of the human upper limb [24], as illustrated in Figure 5, the elbow flexor and extensor primary include 7 parts of muscles. In this paper, a three-dimensional MSM of the human upper limb was established based on public data of skeletal coordinates and muscle architecture [22, 23] and by using the obstacle-set method [25] to model the muscle path. The MSM consists of 3 bones, 3 joints, and 7 parts of muscles.  Table 1 shows the architectural properties of each muscle or muscle part. Additional details regarding the MSM are available in the references mentioned above [22, 23].

#### 2.3.1. Muscle Geometry

The obstacle-set method [25] was used to model the joint configuration-depended muscle path. This method uses some regular-shaped rigid bodies, like a cylinder, to serve as obstacles fixed on and move with the skeleton to force muscles wrap on it for all joint configurations. In the obstacle-set method, the muscles were treated as mass-less, friction-less cables that follow the shortest path between the origin point and insertion point. The action line of MTF is determined by fixed or obstacle via points, origin, and insert points. Garner [25] presented the detailed descriptions of the algorithms and formulas about this method.

**Figure 6:**
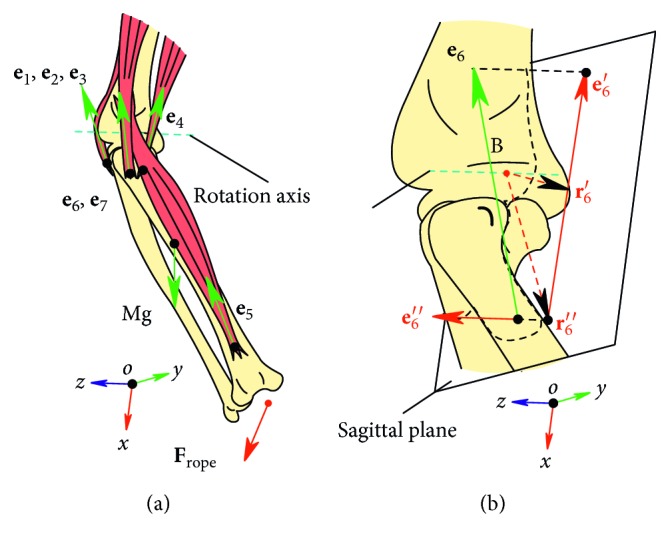
(a) Force sharing of MTF across elbow joint. **e**_*i*_(*i* = 1 – 7) denotes the action line of MTF; (b) the action line of **e**_6_ was projected into sagittal plane (**e**_6_′) and along rotation axis (**e**_6_″). **r**_6_′ and **r**_6_″ are their moment arm.

In the obstacle-set method mentioned above, the shortest path of muscle wrapping is computed analytically and muscle-tendon length is calculated as the sum of the straight-line segments and wrapping segments. Different from the tendon displacement method [26], we classically defined moment arm as the distance between muscle's action line and joint's axis of rotation [27]. The MSM is a detailed three-dimensional model, and the action line of muscles is usually not in the sagittal plane. As illustrated in Figure 6(b), we project their action line into the sagittal plane to calculate moment arm based on geometric calculation.

#### 2.3.2. Optimization Process

As illustrated in Figure 6(a), the human musculoskeletal system is usually characterized by redundant muscles, and load sharing is closely related to the action line of MTF and the rotation axis. The static optimization method is usually used to solve this redundant problem. The static optimization is a computationally efficient method used in predicting redundant MTF by minimizing a cost function subject to force/torque constraints associated with a given task [28, 29]. Equilibrium equations include components in the sagittal plane, and along the rotation axis, two constraint equations were constructed for optimization. MTF is also constrained between zero and maximum MTF by an inequality constraint. The objective function is expressed as the sum of muscle stress squared. Gravity of the forearm is another contributor to the resultant moment about the elbow joint. Static optimization is formulated as follows:

(5) $$\begin{array}{c} \text{minimize}\;\overset{n}{\underset{i=1}{\sum }}{\left(\frac{F_{i}^{\text{mt}}}{A_{i}}\right)}^{2},\\ \text{subject to}\;\left\{\begin{array}{c} \overset{n}{\underset{i=1}{\sum }}F_{i}^{\text{mt}}\cdot r_{i}^{\prime }\times e_{i}^{\prime }+{Μ}^{\prime }=\left[I\right]\cdot \alpha ,\\ 0\leq F_{i}^{\text{mt}}\leq F_{0i}^{\text{M}}, \end{array}\right. \end{array}$$

where *F*_*i*_^mt^ is the magnitude of MTF; *A*_*i*_ is the physiological cross-sectional area (PCSA); **e**_**i**_′ is the sagittal projection of the action line and **e**_**i**_′ is the sagittal moment arm; **Μ** is the resultant joint moment of gravity, resistance force, passive muscle fiber force, and joint reaction moment and **Μ**′ is its projection in the sagittal plane; [**I**] is the inertia mass matrix of the forearm; **α** is the angular acceleration at the elbow joint (in this study, angular acceleration is relatively small and is assumed as 0); and *F*_0*i*_^M^ is the maximum isometric muscle fiber force.

### 2.4. Estimation of Muscular States

#### 2.4.1. Muscle-Tendon Model

A Hill-type muscle model was utilized to represent the intrinsic mechanical properties of human muscles. Each musculotendon actuator is represented as a 3-element muscle in series with an elastic tendon. The instantaneous length of the actuator is determined by the length of the muscle, the length of the tendon, and the pennation angle of the muscle. In this model, the pennation angle is assumed to remain constant as muscle length changes [30].

For a specific muscle *i*, general form of the function of the Hill-type muscle model is given by the following equation:

(6) $$\begin{array}{c} F^{\text{mt}}\left(t\right)=F^{t},\\ =\left[{F_{\text{A}}}^{\text{m}}+{F_{\text{P}}}^{\text{m}}\right]\cos\left(\varphi \right),\\ =\left[f_{\text{A}}\left(l\right)f\left(v\right)a\left(t\right){F_{\text{0}}}^{\text{M}}+f_{\text{P}}\left(l\right){F_{\text{0}}}^{\text{M}}\right]\cos\left(\varphi \right), \end{array}$$

where *F* ^mt^(*t*)=*F* ^*t*^ is the time-varying MTF; *F*_A_^m^ and *F*_P_^m^ are the active muscle fiber force and passive muscle fiber force; *l*  = *l* ^m^ */l*_0_^m^ is the normalized muscle fiber length; *v*=*v* ^m^/*v*_0_^m^ is the normalized fiber velocity; *l*_0_^m^ is the optimal fiber length; *v*_0_^m^ is the maximal fiber velocity; *a*(*t*) is the time-varying muscle activation; *φ* is the muscle pennation angle; *f*_A_(*l*) and *f*_P_(*l*) are the normalized active and passive force-length relationships; and *f*(*v*) is the normalized curve of the velocity-dependent muscle fiber force. *f*_A_(*l*), *f*_P_(*l*), and *f*(*v*) are the nonlinear formulas that characterize the material properties of the muscle tissue. In this model, we use the curves created by cubic spline interpolation of points defined on the Gordon Curve [31, 32]. The curves were normalized for force, length, and velocity. The maximum muscle fiber contraction velocity of all muscles was assumed to be *v*_0_^m^ = 10*l*_0_^m^ [33].

#### 2.4.2. Estimating Muscular States

According to (6), the muscle fiber length and fiber velocity are needed in estimation of passive and active muscle fiber force. For a specific muscle, we approximate the muscle-tendon length as a function of *θ*:

(7) $$\begin{array}{c} l^{\text{mt}}=\Psi_{4}\left(\theta \right). \end{array}$$

The muscle-tendon length includes two parts: tendon length *l* ^t^ and fiber length *l* ^m^:

(8) $$\begin{array}{c} l^{\text{mt}}=l^{\text{t}}+l^{\text{m}}\cos\left(\varphi \right). \end{array}$$

**Figure 7:**
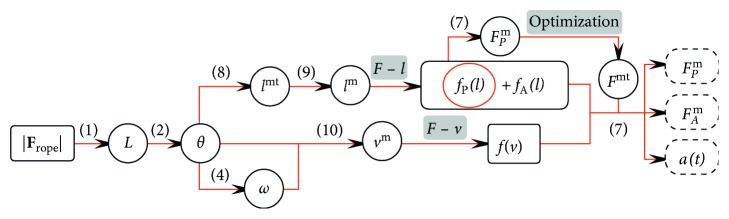
Block-diagram of the algorithm used to estimate muscular states.

Suppose the change of muscle-tendon length is mainly the result of the change of fiber length, we have

(9) $$\begin{array}{c} \frac{dl^{\text{mt}}}{dt}=\frac{d\Psi_{4}\left(\theta \right)}{dt}=\Psi_{5}\left(\theta ,w\right)=-v^{\text{m}}\cos\left(\varphi \right), \end{array}$$

where *v* ^m^ is the fiber velocity and *v* ^m^>0 means the muscle is shortening and *v* ^m^<0 means the muscle is lengthening.  Figure 7 illustrates the algorithm used in the estimation of muscular states.

## 3. Results

### 3.1. Testing of the Measuring System

**Figure 8:**
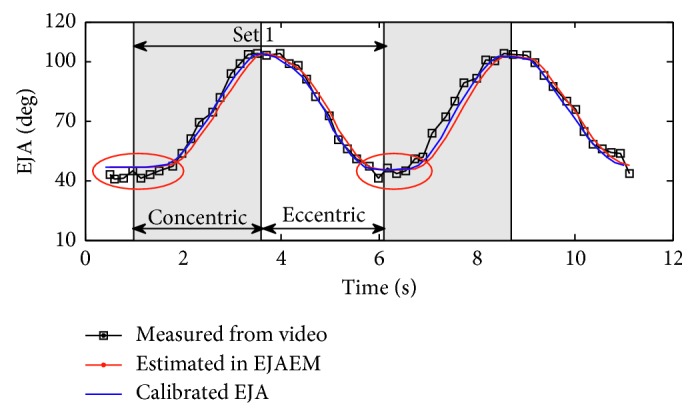
Comparison of elbow joint angle measured from video and that estimated in EJAEM.

Contrasting experiments were carried out to test the correctness of the measuring system in estimating elbow joint angle. In the experiment, a subject was asked to perform two sets of concentric and eccentric contractions in flexor resistance training (the experiment was conducted with the subject's understanding and consent). Initial configurations of the training setup were measured using rulers and typed into the model (*S*_1_ = 1080 mm, *S*_2_ = 1384 mm, *L*_1_ = 270 mm, *L*_2_ = 304 mm, *L*_0_ = 870 mm, and *H* = 230 mm). We recorded the video and measured the real elbow joint angle by using a protractor and compared it with that estimated in EJAEM. As we can see from the results illustrated in Figure 8, the elbow joint angle estimated in EJAEM shows good consistency with that measured from the video except when the angle is less than the initial angle *θ*_0_. This is because when |**F**_rope_| is zero, we set the rope length at its initial length in the program. Calibration was performed to eliminate the time delay caused by software and hardware. The good consistency between measured and estimated result demonstrates that this measuring system can correctly estimate elbow joint angle when the forearm flexes or extends in the sagittal plane.

### 3.2. Evaluation of the Musculoskeletal Model

**Figure 9:**
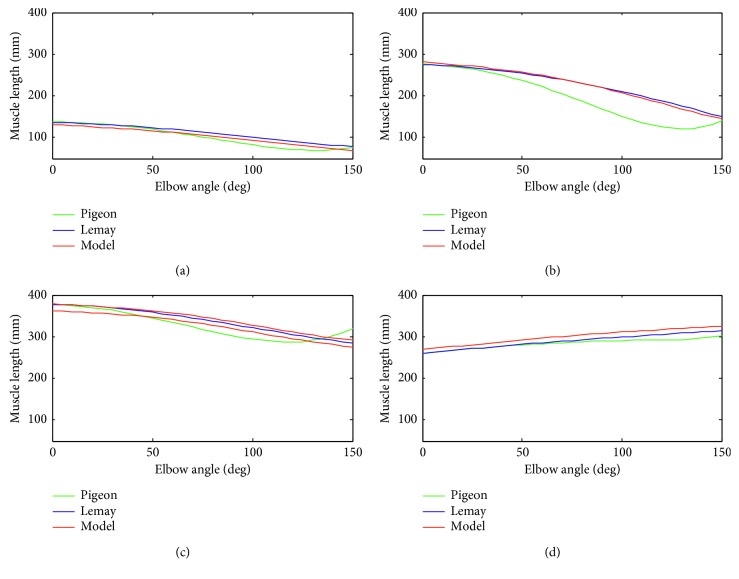
Comparison of muscle length as a function of elbow joint angle. (a) BRA (b) BRD (c) BIC (d) TRI.

**Figure 10:**
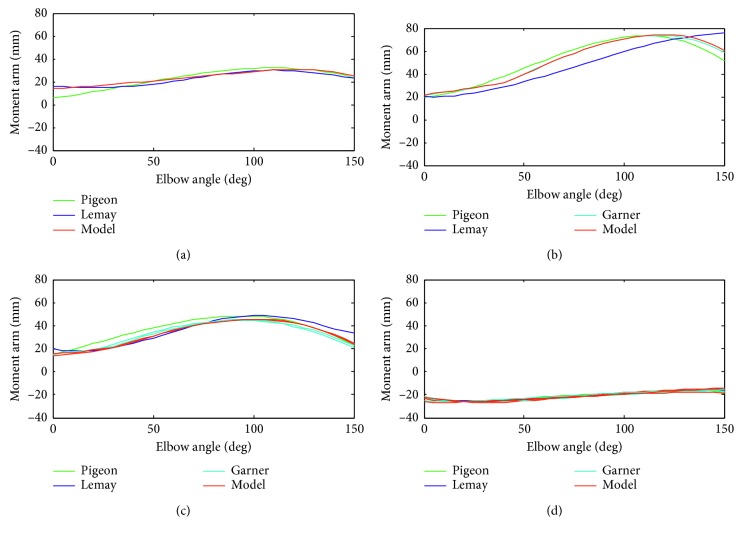
Comparison of moment arm as a function of elbow joint angle. Positive values indicate flexion moment arm and negative values indicate extension moment arm. 0° means the elbow is fully extended. (a) BRA (b) BRD (c) BIC (d) TRI.

The MSM provided by Garner [22, 23] is a detailed three-dimensional model of the human upper limb. But, the EJAEM in this paper is a simplified two-dimensional model in the sagittal plane. Therefore, we projected muscles into the sagittal plane, calculated muscle length and moment arm, and compared them with results provided by Garner [34], Lemay [35], and Garner [23] as an evaluation of the MSM. Their results were obtained by using anatomical or experimental data. As illustrated in Figures 9 and 10, muscle length and moment arm were calculated as a function of elbow joint angle with the shoulder joint at neutral position, the humerus in parallel with the *y* axis of the thorax, and elbow joint angle varying from 0° to 150°. We used the polynomial coefficients of muscle length and moment arm provided by Pigeon and Lemay, which were approximated by using anatomical or model data, and point data of moment arm illustrated in Garner [23]. Lemay only provided the length changes of the muscle, so we use the constant portion of muscle length provided by Pigeon [34]. The muscle length and moment arm estimated in this model demonstrate a substantial agreement with that provided in their documents, especially the moment arm compared with Garner. The data used in this model originate from the study of Garner, but we use a different method in calculating the moment arm. Garner calculated the moment arm by computing the derivative of muscle length with respect to joint angle [24]. Good coherence of muscle length and moment arm demonstrates that the established MSM captures important mechanical features of muscles across elbow joints and is adequate to serve as a generic model to analyze muscle kinematics in the case of elbow flexing or extending.

### 3.3. Muscular States and Mechanical Stimuli during Flexor Resistance Training

**Figure 11:**
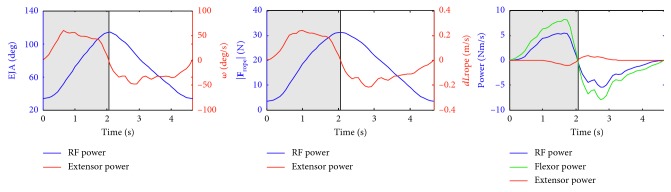
The elbow joint angle (EJA), angular velocity (*ω*), resistance force (|**F**_rope_|), length change rate (*dL*_rope_), power of resistance force, power of flexor, and extensor in the training.

**Figure 12:**
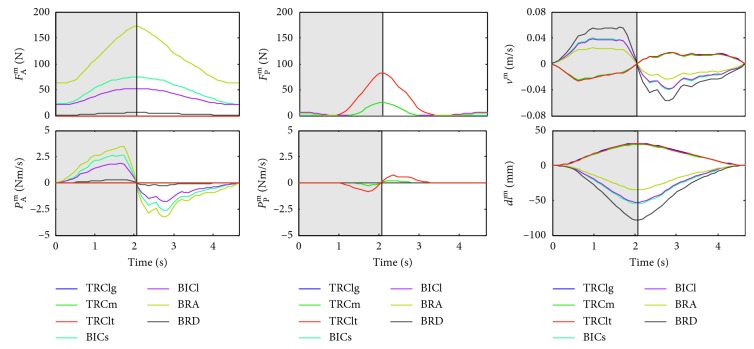
The active (*F*_A_^m^) and passive (*F*_P_^m^) muscle fiber force, muscle fiber velocity (*v* ^m^), power of active (*P*_A_^m^) and passive (*P*_P_^m^) muscle fiber force, and length changes of muscle fiber in the training.

The muscular state refers to mechanical variables such as the muscle-tendon length changes and the muscle fiber velocity. Mechanical stimuli such as power and work are derivative of those mechanical variables. One set of concentric and eccentric contraction was performed by a healthy subject in flexor resistance training and data were utilized to show body-level and muscle-level stimuli in Figures 11 and 12 and in Table 2. *ω*, *dL*_rope_, and *v* ^m^ were the derivative of *θ*, rope length, and fiber length versus time, respectively; power was the product of force and velocity; work was the accumulation of the product of force and displacement over each time step; flexor and extensor power was calculated as the sum of power of flexor and extensor. Table 2 shows the work and displacement, as well as the average force of resistance force and muscles. Work is the accumulation of the product of muscle fiber force and displacement over each time step. Length change is the accumulation of small displacement over each time step, and average force is the mean value of force.

The calculated results show important characteristics about the mechanical stimuli during flexor resistance training. In concentric contraction, resistance force reaches its maximum within two seconds and the maximal angular velocity is about 50 deg/s. Most of the muscle-level stimuli show good consistency with the body-level stimuli. For example, near the maximal elbow joint angle, the resistance force, active and passive muscle fiber force, and length change of muscle fiber reach their maximum or minimum at the same time and their curves show a good consistency. But because of the differences in muscle architecture, the muscle fiber velocity shows a significant difference between flexor and extensor, and the curves of muscle fiber velocity are different from the curve of angular velocity. The resistance force power can be considered as the combined effect of flexor and extensor power. Passive muscle fiber force, which appears when the fiber length exceeds its optimal fiber length, affects active muscle fiber force of other muscles. BRA was the biggest energy provider and produced the biggest average and maximal force; this is probably because BRA possesses the biggest PCSA among flexor.

## 4. Discussion

**Figure 13:**
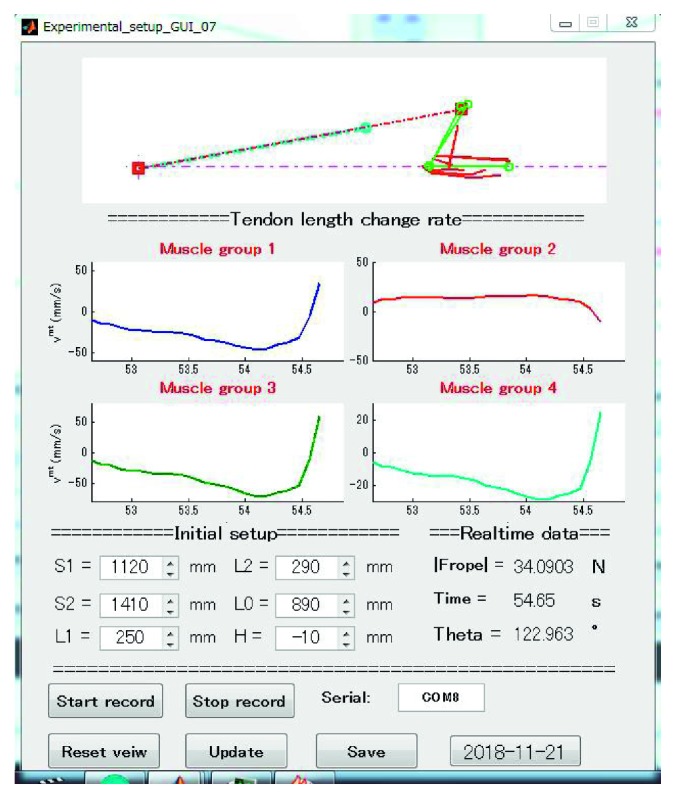
The GUI which is built to show the MSM, muscle velocity (*v* ^mt^), initial setup, and some real-time results. A subject was extending his forearm and the muscle velocity changes over time were showed to the users (muscle velocity reflects the state of muscle shortening and stretching. Less than 0 means muscle shortening, and more than 0 means muscle stretching). Muscles were divided into four groups, and the muscle velocity of each group is the average muscle velocity. BICS and BICl belong to Group 1. TRClg, TRClt, and TRCm belong to Group 2. BRD belongs to group 3, and BRA belongs to group 4.

Simplicity and usefulness are two important features of the system. The only training device required in this system is a fitness tube with a load cell which was designed to measure the resistance force. Combining the resistance force with the established models, the mechanical states of muscles can be roughly estimated and monitored during the training. Many studies reveal that muscle velocity and muscle power (the product of force and velocity) are critical determinants of physical functioning in older adults [10], and the velocity loss is an indicator of neuromuscular fatigue during resistance training [36]. As shown in Figure 13, we built a GUI to help the users type in the initial setup of the experiment and interact with the MSM. The real-time interaction makes the MSM like a sensor which can be used to measure muscle kinematic parameters such as muscle length changes and muscle velocity (*v* ^mt^). Through the GUI, the patient can see the velocity change of his muscle and choose the appropriate training dose and intensity based on his feeling or the instruction of the physiotherapist. Visual interaction increases the patient's interest in the training process. Maximum muscle velocity and other mechanical stimuli (such as maximum RF, power, and work) can be used as relative indicators for recording the training phase or setting training goals.

This study presents some limitations. Due to the intrinsic property of static optimization, the optimized MTF is closely related to the objective function and constraint conditions. Without changing the muscle architecture in the model, experiments were conducted on two other participants with different heights and weights. The calculation results show that the measurement system can correctly estimate the angle of the elbow joint, but the muscle activation patterns are almost the same when the elbow flexes and extends. Differences are the measured resistant force and angular velocity of the forearm. However, the generic MSM is sufficient to provide reliable indicators to record relative changes of training intensity at different training stages. Comparison between estimated force and surface electromyographic signals of muscle is planned for future work to show the extent to which the optimized MTF reflects the actual muscular states. And because the elbow flexing and extending was limited in the sagittal plane, the muscle activation patterns are relatively simple in the movement. Future work also needs to enhance the system to include more degrees of freedom in the MSM and apply it to other types of resistance exercises like elbow extensor resistance training and pull-ups.

## 5. Conclusion

This paper presents the concept of using musculoskeletal modeling to estimate muscular states during elbow flexor RT for bedridden patients, and it is mainly on the discussion of computational methods. We take the elbow flexor RT as a simple example, and an integrated system was built for this exercise. The design concepts of the system, the measurement, and analysis methods were described in detail. We recorded the video about the training process and measured the real elbow joint angle by using a protractor and compared it with that estimated in EJAEM. The results demonstrate that the measuring system can correctly estimate the elbow joint angle when the forearm flexes or extends in the sagittal plane. The muscle length and muscle moment arms were calculated and compared with results provided in other references to show that the established MSM is adequate to serve as a generic model to analyze muscle kinematics in the case of elbow flexing or extending in the sagittal plane. The system offers a simple method to monitor muscle states during elbow flexor RT in bedridden patients, providing coaches or physiotherapists with practical muscle-related information to evaluate the training process. The calculations also demonstrate that the musculoskeletal modeling is a considerable method to vividly analyze the muscular states during training.

## Figures and Tables

**Table 1:** Architectural properties of each musculotendon actuator of elbow extensor and flexor.

Muscles		Abbr.	PCSA (cm^2^)	*l*_0_^m^ (N)	*v*_0_^m^ (cm/s)	lSt (cm)	*F*_0_^M^ (N)	*φ* (deg)
Extensor	1. Triceps brachii (long)	TRClg	19.07	15.24	152.4	19.05	629.21	15.00
	2. Triceps brachii (lateral)	TRClt	38.45	6.17	61.7	19.64	1268.87	15.00
	3. Triceps brachii (medial)	TRCm	18.78	4.90	49.0	12.19	619.67	15.00

Flexor	4. Brachialis	BRA	25.88	10.28	102.8	1.75	853.90	15.00
	5. Brachioradialis	BRD	3.08	27.03	270.3	6.04	101.58	5.00
	6. Biceps brachii (long)	BICl	11.91	15.36	153.6	22.93	392.91	10.00
	7. Biceps brachii (short)	BICs	13.99	13.07	130.7	22.98	461.76	10.00

**Table 2:** Work, maximal change in length, and average and maximal force of resistance force and muscles. We set the direction of elongation along the rope or muscle path as the positive direction of force and length change.

	|**F**_rope_|	Extensor			Flexor			
		TRClg	TRCm	TRClt	BICs	BICl	BRA	BRD
Work (N∗m)	−6.24	0	−0.105	−0.502	3.06	2.02	4.03	0.308
Maximal change (mm)	316.04	31.61	30.13	31.34	−55.08	−52.99	−34.89	−78.10
Average force (N)	−17.34	0	−4.98	−20.19	−49.58	−36.62	−109.12	−3.43
Maximal force (N)	−31.27	0	−26.21	−82.76	−74.13	−52.42	−171.93	−5.94

## Data Availability

The data used to support the findings of this study are available from the corresponding author upon request.

## Abbreviations

EJAEM: Elbow joint angle estimation model
MSM: Musculoskeletal model
MTF: Muscle-tendon force
MTM: Muscle-tendon model
RT: Resistance Training.

## Supplementary Materials

Supplementary Materials Supplementary material file for the video clip about the GUI of the integrated system in which a healthy subject was asked to perform two sets of concentric and eccentric contractions and some real-time results were displayed to audience via the GUI. (Supplementary Materials)
